# Exploring the Mechanism of Skeletal Muscle in a Tacrolimus-Induced Posttransplantation Diabetes Mellitus Model on Gene Expression Profiles

**DOI:** 10.1155/2020/6542346

**Published:** 2020-01-09

**Authors:** Chenlei Zheng, Cheng Wang, Tan Zhang, Ding Li, Xiao-feng Ni, Jian-Hu Lin, Linxiao Sun, Bicheng Chen

**Affiliations:** Key Laboratory of Diagnosis and Treatment of Severe Hepato-Pancreatic Diseases of Zhejiang Province, Zhejiang Provincial Top Key Discipline in Surgery, The First Affiliated Hospital of Wenzhou Medical University, Wenzhou, Zhejiang, China

## Abstract

**Objective:**

Posttransplantation diabetes mellitus (PTDM) is a known complication of transplantation that affects the prognosis. Tacrolimus (Tac or FK506) is a widely used immunosuppressant that has been reported to be a risk factor for PTDM and to further induce complications in heart and skeletal muscles, but the mechanism is still largely unknown. In our preliminary experiments, we found that after Tac treatment, blood glucose increased, and the weight of skeletal muscle declined. Here, we hypothesize that tacrolimus can induce PTDM and influence the atrophy of skeletal muscle.

**Methods:**

We designed preliminary experiments to establish a tacrolimus-induced PTDM model. Gene expression profiles in quadriceps muscle from this rat model were characterized by oligonucleotide microarrays. Then, differences in gene expression profiles in muscle from PTDM rats that received tacrolimus and control subjects were analyzed by using GeneSpring GX 11.0 software (Agilent). Functional annotation and enrichment analysis of differentially expressed genes (DEGs) helped us identify clues for the side effects of tacrolimus.

**Results:**

Our experiments found that the quadriceps in tacrolimus-induced PTDM group were smaller than those in the control group. The study identified 275 DEGs that may be responsible for insulin resistance and the progression of PTDM, including 86 upregulated genes and 199 downregulated genes. GO and KEGG functional analysis of the DEGs showed a significant correlation between PTDM and muscle development. PPI network analysis screened eight hub genes and found that they were related to troponin and tropomyosin.

**Conclusions:**

This study explored the molecular mechanism of muscle atrophy in a tacrolimus-induced PTDM model by bioinformatics analyses. We identified 275 DEGs and identified significant biomarkers for predicting the development and progression of tacrolimus-induced PTDM.

## 1. Introduction

Posttransplantation diabetes mellitus (PTDM) is a known complication of transplantation [1]. It has been reported to be associated with cardiovascular disease, skeletal muscle dysfunction, graft failure, and the length of patient survival [2–5]. PTDM may also affect the long-term outcomes of transplant patients. Early detection of PTDM risk factors and PTDM management are concerns of posttransplantation care. Several risk factors for PTDM have been reported, including age, body mass index, hepatitis C infection [6–10], and immunosuppressive medication [7, 11].

Tacrolimus (Tac or FK506) has been reported as a risk factor for PTDM because it can cause insulin hyposecretion [5, 7, 12]. A previous study reported that during the first 5-month Tac treatment period, rats suffer from decreased insulin secretion, inducing increase insulin resistance, and undergo islet cell injury [13]. Impaired energy metabolism in skeletal muscle occurs with the development of diabetes mellitus [14]. It has been reported that DM induces muscle atrophy and transformed fiber type from oxidative to glycolytic [15–17]. However, the mechanism remains largely unknown.

Gene expression profiles measure the expression of thousands of genes simultaneously to build a global picture of cell function, and gene expression profiling can be used as an important diagnostic test [18, 19]. This technique may have positive influence to improve our knowledge of transplantation and facilitate the screening and early detection of complications.

Therefore, we hypothesize that tacrolimus induces PTDM and affects skeletal muscle function such as atrophy. It has been reported that quadriceps are kinds of oxidative muscle which were closely related to insulin resistance [20] while the muscle fiber types of gastrocnemius are glycolytic type [20]. Our preliminary study had shown peroxisome proliferator-activated receptor- (PPAR-) *γ* which was related to insulin resistance in Tac-induced PTDM expressed in quadriceps [21]. So, quadriceps and gastrocnemius were dissected from the PTMD model induced by Tac to test this hypothesis. In this work, we characterized gene expression profiles of muscle from a rat model using oligonucleotide microarrays. DEGs between tacrolimus-induced PTDM model tissues and normal tissue samples were identified through gene expression profiling. In the end, several candidate genes were identified to be responsible for the progression of PTDM and effects on skeletal muscle. Our results offer information on this PTDM model and provide potential therapeutic targets for further studies.

## 2. Materials and Methods

### 2.1. Material

Tacrolimus capsules, approval number: Zhunzi J20090142, were purchased from Astellas Pharma Co., Ltd. (China); saline was purchased from Haili Chen Biotechnology Co., Ltd.; chloral hydrate was purchased from Shanghai to Beijing Biological Technology Co., Ltd. Cable; a blood glucose meter and blood glucose test strips were purchased from Changsha Sannuo Biosensing Co.; and TRIzol reagent was purchased from Gibco BRL.

### 2.2. Preliminary Study

We designed preliminary experiments to establish the maximum dose of tacrolimus needed to control the clinical conditions of most of the patients to determine the target trough plasma levels of tacrolimus (≤20 ng/mL). We treated three groups of animals with i.p. tacrolimus (0.1, 1, and 10 mg/kg/day). Animals were treated for 7 days, and plasma levels of the drug were serially measured. The target trough level of tacrolimus was achieved with a dose of 1 mg/kg/day.

### 2.3. Animal Treatment to Establish the PTDM Rat Model

Male Sprague-Dawley rats (body weight: 200-250 g) were obtained from Vital River Laboratory Animal Technology Co. Ltd, housed in cages at 22°C under a 12 h light-dark cycle, and fed commercial pellet chow ad libitum. All animal care, use, and experimental protocols were submitted and approved by The Tab of Animal Experimental Ethical Inspection of Laboratory Animal Centre, Wenzhou Medical University. Groups of rats were injected once a day with saline (control) or FK506 in saline (1 mg/kg BW). Body weight and glucose, which was measured in the tail blood with an Accu-chek Sensor instrument, were regularly recorded every two days at the end of the light period. Once the treatment finished, animals were euthanized and their quadriceps and gastrocnemius were dissected out.

### 2.4. Histopathological Analysis

The quadriceps and gastrocnemius of both groups were collected and weighed and then fixed in paraformaldehyde for 36 h. The tissues were dehydrated in graded ethanol solutions, vitrified with dimethylbenzene, and embedded in paraffin. Paraffin-embedded muscles were cut into 4.5 *μ*m sections and then deparaffinized and rehydrated. The muscles were stained with hematoxylin-eosin (HE; Solarbio) and observed with a light microscope at 200x. The cross-section area was determined for at least 100 fibers per tissue.

### 2.5. RNA Isolation and Microarray Analysis

This dataset included 6 samples of quadriceps muscle from PTDM rats that received tacrolimus and normal rats with three biological replicates. RNA was extracted from the samples with TRIzol reagent according to the manufacturer's protocol. RNA was reverse transcribed by a RevertAid First Strand cDNA Synthesis Kit. Further labeling and microarray hybridization experiments and washes were performed at GeneChem (Shanghai, China). Microarray analysis was performed on an Agilent platform (Agilent Technologies, USA). Differentially expressed genes in PTDM rats were defined as follows: (1) normalized intensity: after normalizing with median scaling normalization method, take the average of repeated data from the same sample. The software of Rosetta Resolver was used to list normalized data (2) *p* value < 0.05 and minimum fold change > 1.5 (absolute value of log2 ratios is ≥0.585) using GeneSpring GX 11.0 software (Agilent) and (3) a difference of intensity between the two groups ≥ 1000 when the log2 ratios = NA.

### 2.6. Functional Analysis of DEGs

GO and KEGG enrichment analysis of differentially expressed genes in skeletal muscle from PTDM rats was performed using WebGestalt. Web Gene Ontology Annotation Plot (WEGO 2.0) was used to visualize the GO classification results. The STRING database (https://string-db.org) is a system for determining interactions between genes or proteins and was used to guide the construction of the PPI network of downregulated top 50 DEGs to explore functional interactions [22]. Parameters used to retrieve the information from STRING were default. Only nodes with combined score > 0.900 and node degree ≥ 1 were included in the PPI networks. Experiments and databases were chosen as active interaction sources.

### 2.7. Quantitative Real-Time PCR (qRT-PCR)

The RNA expression of Tpm3, Tnnc1, Tnnt1, Tnni3, Hrh3, Apln, S1pr3, and Cxcl12 was detected by qRT-PCR. The experiment was performed with FastStart Universal SYBR Green Master (ROX) using a machine of 7500 Fast machine (Applied Biosystems). GAPDH was used as an internal standard, and the results were analyzed by the 2^-*ΔΔ*Ct^ method. The sequences of the primers used are listed in Table 1.

### 2.8. Statistical Analyses

All data are presented as mean values ± SD and performed using one-way ANOVA. *p* values < 0.05 were considered statistically significant.

## 3. Results

### 3.1. Establishment of a Rat Model of PTDM

**Figure 1:**
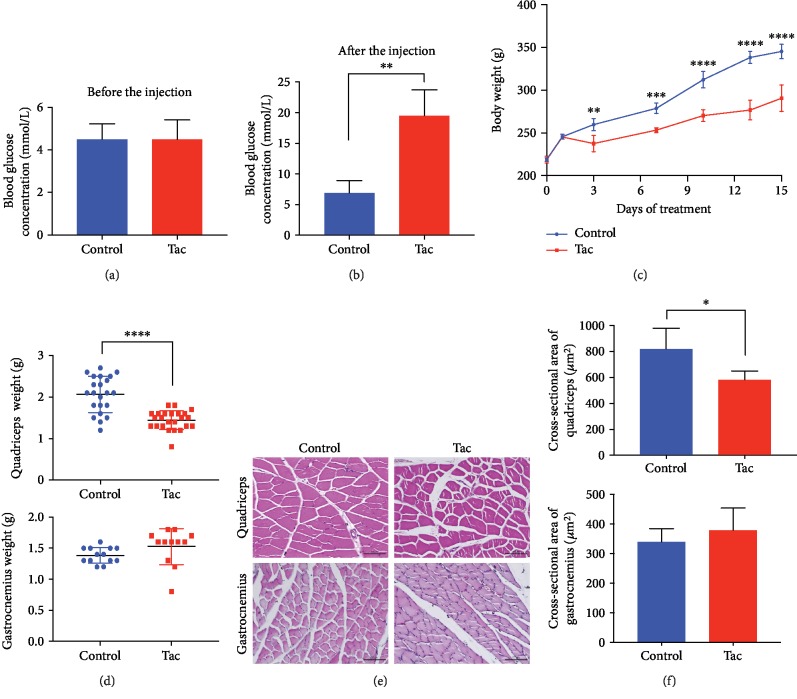
Skeletal muscle atrophy in rats with posttransplantation diabetes mellitus (PTDM). Blood glucose concentrations before and after the injection (a, b). Body weight of the rats (c). Wet weight of quadriceps and gastrocnemius (d). Cross-section area of quadriceps and gastrocnemius stained with hematoxylin and eosin (e). The data of cross-section area (f). Scale bars represent 50 *μ*m. Data are means ± SD. ∗*p* < 0.05, ∗∗*p* < 0.01, ∗∗∗∗*p* < 0.0001.

The PTDM rats were treated as described. The blood glucose concentrations of the PTDM and control groups were 4.48 ± 0.92 and 4.49 ± 0.73 mmol/L, respectively (*p* > 0.05), before the injection. After two weeks of treatment, the blood glucose concentrations of the two groups were 19.51 ± 4.22 and 6.87 ± 2.05 mmol/L, respectively, and the concentration of the PTDM group was significantly higher than that of the control group (*p* < 0.01) (Figures 1(a) and 1(b)). The body weight of the rats increased in both groups during the study, and the control group weighted more heavier than the Tac group (*p* < 0.0001) (Figure 1(c)). The wet weight of quadriceps in the Tac group was lighter than that in the control group (*p* < 0.0001) (Figure 1(d)). The cross-section area of pathological section in quadriceps showed that the Tac group was smaller than the control group (*p* < 0.05). Those gaps between muscle fibers were narrow in the control group. Fibers of quadriceps obtained from Tac rats exhibited atrophy and disordered arrangement (Figures 1(e) and 1(f)). The weight and cross-section area of gastrocnemius in the control and Tac groups were not significantly different (Figures 1(d)–1(f)).

### 3.2. Differential mRNA Expression in Muscle from PTDM Rats

**Figure 2:**
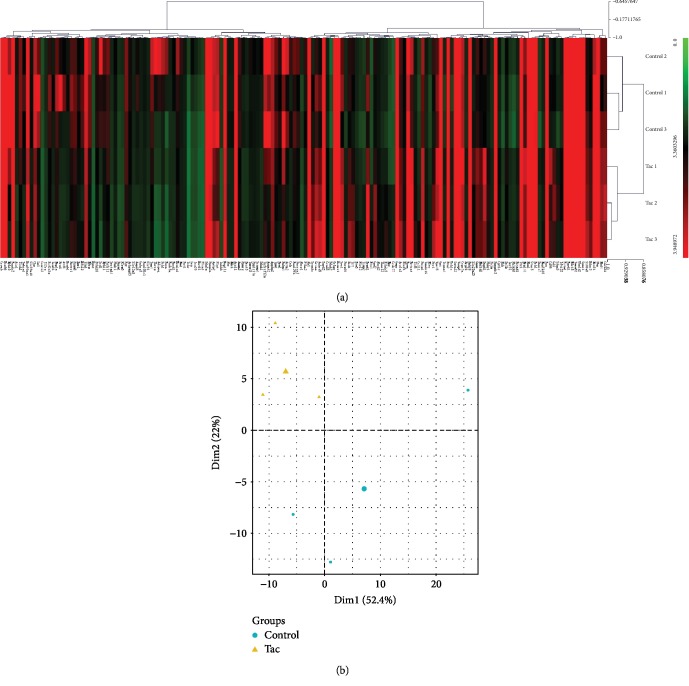
Differentially expressed genes profiles. Heat map of DEGs in Tac rats and controls (a). Principal component analysis (PCA) of DEGs (b).

The quality of the samples is shown in Table 2. Background intensity in the channel (Cy5) of the two groups is <200. About the internal control gene, mean of IHC intensity is >37500. ETQC, labeled-spike-in hybridization controls with low, median, and high concentrations, represents a concentration-dependent intensity gradient. The range of ETQC median of six simples was between 70600 and 74995. The quality control results show that all of the samples pass the inspection standard. Heat map analysis (Figure 2(a)) demonstrated differences in mRNA expression levels between the PTDM and control groups considering a *p* value cutoff of 0.05 and a fold change cutoff of 1.5 (upregulated or downregulated). According to the threshold in this study, we identified 275 differentially expressed genes, 86 upregulated genes and 199 downregulated genes, between these two groups. The majority of differentially expressed genes in muscle from PTDM rats exhibited expression levels with a fold change lower than 4.0 (up or down) when compared to those in muscle from control subjects, and a few genes showed a fold change ≥ 4.0. The principal component analysis (PCA) (Figure 2(b)) score plot of DEGs showed a clear separation between the control and Tac groups, suggesting that Tac has a firm effect on the muscle of rats.

### 3.3. GO Enrichment Analysis of DEGs

**Figure 3:**
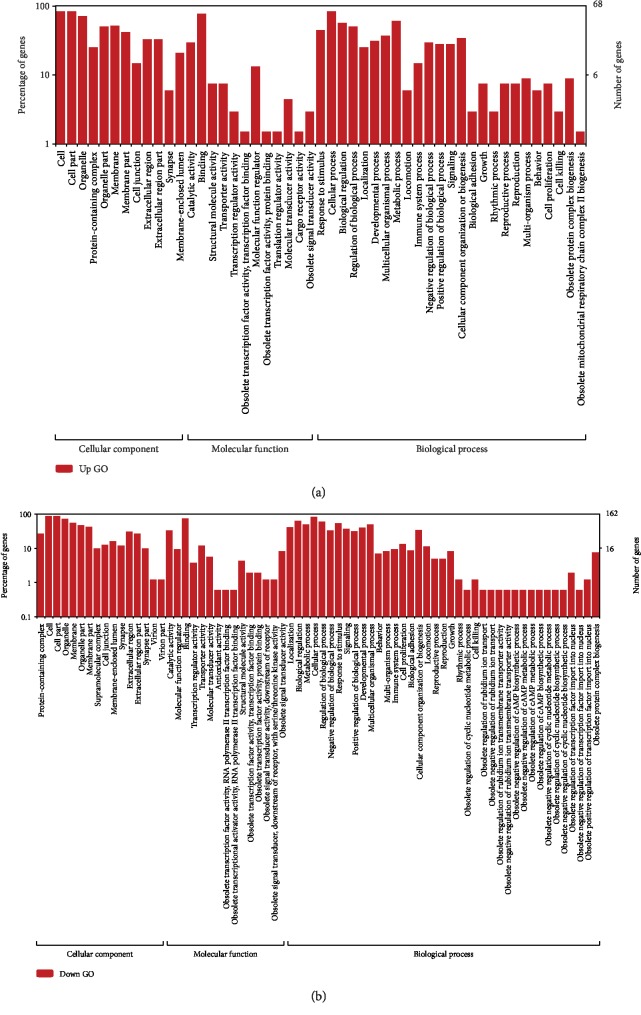
GO classification analysis of upregulated (a) and downregulated (b) DEGs.

To assess the function of the differentially expressed genes in skeletal muscle, we first assigned the genes to different GO levels. The GO classification analysis showed a relatively high abundance of genes involved in binding and cellular processes (Figure 3). Then, we performed enrichment analysis of the cell component, biological process, and molecular function categories and KEGG pathway analysis through WebGestalt with the rat genome as a reference. For the upregulated genes, no significant enrichment items with FDR < 0.05 were found. When we focused on downregulated genes, several significant enrichment items with FDR < 0.05 showed interesting phenomena regarding skeletal muscle (Table 3).

According to GO enrichment analysis, downregulated differentially expressed genes between control and PTDM rats were significantly enriched in multicellular organismal movement, muscle structure development, and circulatory system processes in the biological process category, contractile fibers in the cell component category, and actin binding and cell adhesion molecule binding in the molecular function category. These findings indicated that the GO categories of these differentially expressed probes were predominantly related to muscle function and movement.

### 3.4. KEGG Pathway Analysis of DEGs

**Figure 4:**
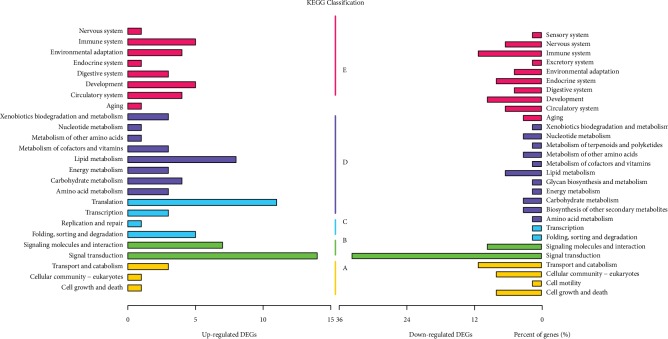
KEGG classification analysis of upregulated and downregulated DEGs. (a) Cellular processes. (b) Environmental information processing. (c) Genetic information processing. (d) Metabolism. (e) Organismal systems.

**Figure 5:**
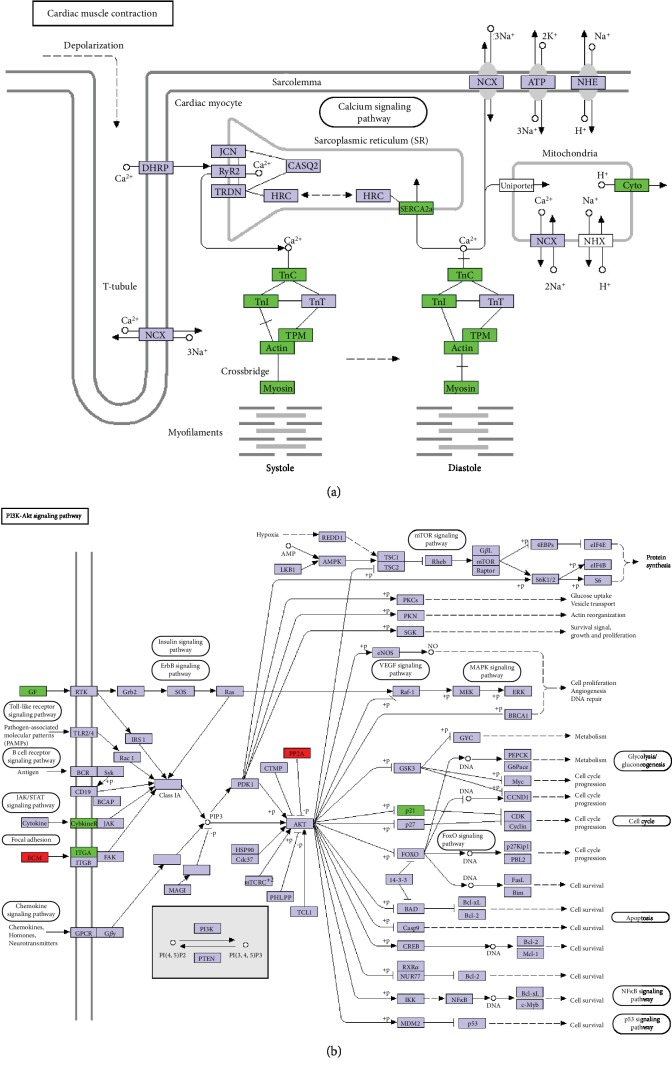
KEGG pathway of cardiac muscle contraction (a) and PI3K-Akt signaling pathway (b). Myofilament is activated by calcium and bind to troponin C. Troponin combines with tropomyosin and actin through the inhibitory protein troponin I without calcium.

The KEGG classification analysis of upregulated and downregulated DEGs was shown in Figure 4. Clearly, the environmental information processing especially signal transduction was significantly higher than other pathways. The classification of translation and replication and repair was related to upregulated DEGs only, while sensory system, excretory system, metabolism of terpenoids and polyketides, glycan biosynthesis and metabolism, biosynthesis of other secondary metabolites, and cell motility are showed in downregulated DEGs. Clearly, the proportion of genes related to signal transduction was significantly higher than that of genes related to other pathways. Interestingly, the cardiac muscle contraction pathway (Figure 5(a)) and calcium signaling pathway (a subset of the signal transduction pathway) had the largest and second largest number of downregulated DEGs, respectively, which indicated that there was a relationship between muscle and PTDM in rats.

Functional enrichment KEGG analysis of downregulated DEGs, as shown in Table 3, showed that the cardiovascular system pathway was significantly differentially expressed between control and PTDM rats. The results showed that the downregulated DEGs participate in muscle contraction and relaxation.

The upregulated DEGs PP2A and ECM as well as downregulated DEGs such as GF, cytokine R, ITGA, and p21 were shown to be involved in the PI3K-Akt signaling pathway (Figure 5(b)). Akt has been reported to be responsible for glucose uptake in skeletal muscle [23].

### 3.5. PPI Network of the DEGs and Hub Genes

**Figure 6:**
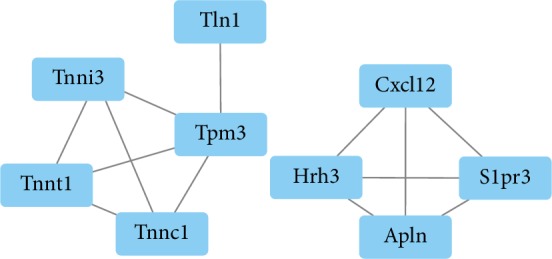
The hub genes were shown in the PPI network.

As seen from the above analysis results, significant functional enrichment was found only in downregulated genes. We further explored whether there was a close significant relationship between the downregulated genes. The PPI network of 41 candidate rat genes obtained by screening for downregulated genes that were significantly enriched in GO and KEGG functions is shown in Figure 6.

**Figure 7:**
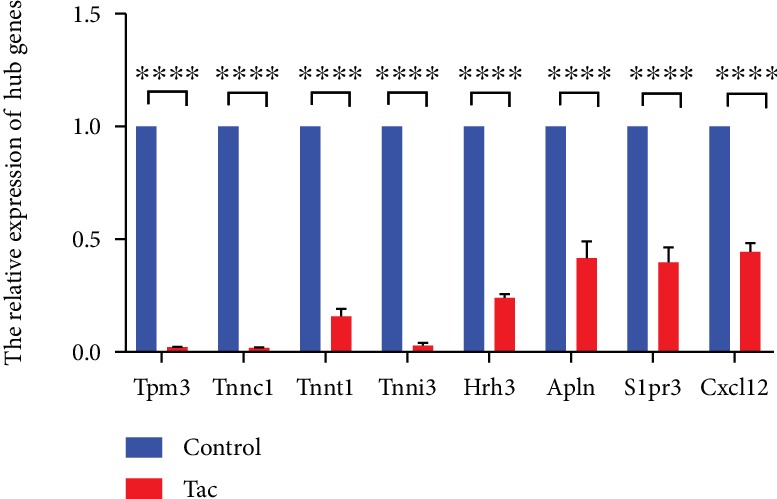
The relative expression of hub genes. Data are means ± SD. ∗∗∗∗*p* < 0.0001.

A PPI enrichment *p* value < 1.0*e*‐16 means that those proteins have more interactions among themselves than what would be expected for a random set of proteins of similar size, drawn from the rat genome. Such an enrichment indicates that the proteins are at least partially biologically connected, as a group. There were 24 nodes and 31 edges in the PPI network based on the top 50 DEGs between two groups, and the average node degree was 2.58. The PPI network contained 8 hub genes according to our criterion, which were Tpm3, Tnnc1, Tnnt1, Tnni3, Hrh3, Apln, S1pr3, and Cxcl12, and all of these genes are related to troponin, myosin, and muscle (Table 4). All of the core genes in the Tac group were validated through the low expression by qPCR (Figure 7).

## 4. Discussion

With the rapid development of the Chinese economy, the number and quality of transplant surgeries have improved dramatically. Immunosuppressive agents such as tacrolimus are necessary for patients to improve the survival of grafts after kidney transplantation. Unfortunately, long-term use of tacrolimus can lead to serious complications, such as posttransplantation diabetes mellitus (PTDM) which is one of the most severe complications of transplantation and influences the survival [24]. Research studies have shown that PTDM increase the risk of cardiovascular events including acute myocardial infarction [25]. DM damages the cardiac contractile and relaxation function and is associated with acute myocardial infarction due to hypertrophied [26, 27]. It has been reported that DM induces skeletal muscle atrophy [15–17], and our research found that Tac-induced PTDM can induce skeletal muscle atrophy. However, the mechanism is not clear, and it is necessary to explore the mechanism of tacrolimus-associated PTDM [28].

A study showed that diabetes mellitus influences the structure and function of skeletal muscle [14, 29]. Skeletal muscle is mainly composed of contractile cells and myofibers and modulates the glucose level in the blood [30]. Myofibers include slow-twitch oxidative fibers (type 1, red muscle) and fast-twitch glycolytic fibers (type 2, white muscle) [31]. Hyperglycemia disrupts the secretion of various angiogenic factors in skeletal muscle cells and influences the survival of skeletal muscle [29]. Thus, skeletal muscle plays an important role in the development of diabetes.

Cardiac muscle contraction is also regulated by troponin and tropomyosin. Troponin is made up of troponin C, troponin I, and troponin T. Under low calcium conditions, troponin combines with tropomyosin and actin through the inhibitory protein troponin I, which leads to the diastole of the myocardium. On the other hand, the activation of myofilament and binding to troponin C are attributed to calcium. The conformational change of troponin inhibits the binding of troponin I and actin, which results in systole [32].

In this study, the microarray method was used to investigate gene expression profiles in Tac-induced PTDM and control subjects. A list of 275 differentially expressed genes (86 upregulated and 199 downregulated) was identified in Tac-treated rats. GO classification analysis showed downregulated expression of DEGs related to muscle structure development. The DEGs were also associated with contractile fibers as a cellular component, actin binding as a molecular function, and so on.

We further built a PPI network of 50 top DEGs, which is shown in Figure 6, and finally identified 8 hub genes: Tpm3, Tnnc1, Tnnt1, Tnni3, Hrh3, Apln, S1pr3, and Cxcl12. Tpm3 (Tropomyosin alpha-3 chain) is an actin-associated protein involved in stress fiber formation [26, 23]. Tropomyosin consists of four genes, including Tpm1, 2, 3, and 4, all of which have different and important functions [33]. Tpm3 promotes the breakdown of glucose and the synthesis of glycogen [23]. The Tnnc1 (Troponin C type 1) gene is a sarcomeric calcium sensor expressed in both slow skeletal and cardiac tissues. Tnnt1 (Troponin T1) is a kind of slow skeletal muscle troponin T (ssTnT) [34]. It is reported that ssTnT deficiency resulted in smaller type 1 fiber [34]. The incapacity of ssTnT to form troponin complex or bind tropomyosin may be the reason for its sensitivity to degradation [35]. It has been reported that the Tnni3 (Troponin I, cardiac muscle) protein changes the activity of actomyosin ATPase, which is involved in the sensing of calcium [36]. Hrh3 (Histamine Receptor H3) was reported to modulate cardiac sympathetic activity and hence ameliorate myocardial remodeling [37]. Apln (Apelin) as a myokine has been used to improve metabolic effects by reducing obesity-related insulin resistance [38–40]. Apelin is associated to mitochondrial function by stimulating Akt through its Gi alpha subunit-coupled receptor APLNR in skeletal muscle cells [41]. S1pr3 (Sphingosine-1-Phosphate Receptor 3) is a target of miR-127, which can enhance myogenic cell differentiation [42]. Cxcl12 (C-X-C Motif Chemokine Ligand 12) promotes the development of myogenesis and regeneration of muscle [43]. Cxcl12 plays an important part in maintaining muscle function ability and homeostasis [44]. The results of this research may help explain the induction of tacrolimus-induced posttransplantation diabetes mellitus via the low expression of muscle genes including Tpm3, Tnnc1, Tnnt1, Tnni3, Hrh3, Apln, S1pr3, and Cxcl12. In the end, these hub gens may serve as new targets for PTDM and provide direction or for clinical treatment.

## 5. Conclusion

Tacrolimus-induced PTDM is a severe complication of posttransplantation with poor prognosis. This study attempted to identify the association between tacrolimus and PTDM by using bioinformatics analysis. Functional analysis of 275 DEGs between the tacrolimus-induced PTDM group and the control group illustrated that muscle atrophy of PTDM is induced by tacrolimus through inhibition of the expression of Tpm3, Tnnc1, Tnnt1, Tnni3, Hrh3, Apln, S1pr3, and Cxcl12. The target genes we found may have a guiding significance for predicting or even reducing the risk to patients. Moreover, the next study will focus on experiments such as western blotting and PCR in clinical cases.

## Figures and Tables

**Table 1:** Sequences of the primers used for quantitative real-time PCR.

Gene	Forward primer (5′–3′)	Reverse primer (5′–3′)
GAPDH	GACATGCCGCCTGGAGAAAC	AGCCCAGGATGCCCTTTAGT
Tpm3	CAGTTCTCCAGTGTTCACAGGTG	TCAGCTCGGTCCAGGACATTCTC
Tnnc1	ACGCTGATGGCTACATTGACTTGG	TCGTTGTTCTTGTCACCGTCCTTC
Tnnt1	CAGGCAGAAGATGAGGAAGCAGTG	CGGAGGAATCAAAGGAGGAACCAC
Tnni3	ACGCTCGTGTGGACAAAGTG	ACTTGCCACGCAGGTCATAG
Hrh3	TCACAGGACAGTCAGCAGGAGAG	ACATTGGCAGAGGCAGCACAC
Apln	TCTGTTCTATTGCCGCTGGTTCAC	GGGCTCTGACTTTCTGTGGGTTG
S1pr3	GGTTGGTGTGCGGCTGTCTG	AGAGTGGCTGCTGCTGTTGTTAC
Cxcl12	TATCTCGGCGGCGTCACCAG	AAACCATCGCTGCGTAGACACTG

**Table 2:** Quality control result.

Item	Background average intensity	Internal control gene	Hybridization control		
	Mean of background intensity (Cy5) < 200	Mean of IHC intensity > 37500	ETQC high	ETQC median	ETQC low
Control 1	110	55446	309703	71525	5099
Control 2	106	62694	295396	70600	5255
Control 3	95	77902	373575	74693	5801
Tac 1	101	48665	319491	73558	5223
Tac 2	98	46770	340991	74995	5517
Tac 3	101	61230	338713	72997	5549

**Table 3:** Functional enrichment analysis of downregulated expression DEGs.

	Term ID	Term	Gene no.	FDR
Biological process	GO:0050879	Multicellular organismal movement	6	0.003027
	GO:0061061	Muscle structure development	16	0.004976
	GO:0003013	Circulatory system process	14	0.006516

Cell component	GO:0043292	Contractile fiber	13	1.31E-05

Molecular function	GO:0003779	Actin binding	13	0.002935
	GO:0050839	Cell adhesion molecule binding	8	0.037984

KEGG	rno04260	Cardiac muscle contraction	7	0.001767
	rno05410	Hypertrophic cardiomyopathy (HCM)	7	0.001767
	rno05414	Dilated cardiomyopathy (DCM)	7	0.001767

**Table 4:** Functional annotation of hub genes.

Gene ID	Gene name	Description
Tpm3	Tropomyosin alpha-3 chain	Binds to actin filaments in muscle and nonmuscle cells
Tnnc1	Troponin C type 1	Slow skeletal and cardiac muscles
Tnnt1	Troponin T1	The tropomyosin-binding subunit of troponin
Tnni3	Troponin I, cardiac muscle	The inhibitory subunit of troponin
Hrh3	Histamine Receptor H3	Increase voltage-dependent calcium current in smooth muscles
Apln	Apelin	Activate specific signaling pathways that regulate insulin secretion
S1pr3	Sphingosine-1-Phosphate Receptor 3	A G-protein-coupled receptor for the bioactive sphingosine-1-phosphate, which is an important regulator of skeletal muscle function
Cxcl12	C-X-C Motif Chemokine Ligand 12	Plays a protective role after myocardial infarction

## Data Availability

All data generated or analyzed during this study are included in this published article and its supplementary information files.

## Supplementary Materials

Supplementary 1 Supplement 1 is the differentially expressed genes including 86 upregulated and 199 downregulated genes of control and PTDM rats with Tacrolimus groups.

Supplementary 2 Supplement 2 is the detail attributes of PPI network.
